# Therapeutic strategies in RET gene rearranged non-small cell lung cancer

**DOI:** 10.1186/s13045-021-01063-9

**Published:** 2021-03-26

**Authors:** Leylah M. Drusbosky, Estelamari Rodriguez, Richa Dawar, Chukwuemeka V. Ikpeazu

**Affiliations:** 1 Guardant 360, 505 Penobscot Drive, Redwood City, CA 94063 USA; 2 Division of Medical Oncology, Department of Internal Medicine, University of Miami Miller School of Medicine, 1475 NW 12th Avenue, Miami, FL 33136 USA; 3 University of Miami Sylvester Comprehensive Cancer Center, 8100 SW 10th Street, Ste 3310F, Plantation, FL 33324 USA

**Keywords:** Non-small cell lung cancer, RET gene fusions, Tyrosine kinase inhibitors, Metastasis

## Abstract

The recent approvals by the Food and Drug Administration several tumor-agnostic drugs have resulted in a paradigm shift in cancer treatment from an organ/histology-specific strategy to biomarker-guided approaches. RET gene fusions are oncogenic drivers in multiple tumor types and are known to occur in 1–2% of non-squamous NSCLC patients. RET gene fusions give rise to chimeric, cytosolic proteins with constitutively active RET kinase domain. Standard therapeutic regimens provide limited benefit for NSCLC patients with RET fusion-positive tumors, and the outcomes with immunotherapy in the these patients are generally poor. Selpercatinib (LOXO-292) and pralsetinib (BLU-667) are potent and selective inhibitors that target RET alterations, including fusions and mutations, irrespective of the tissue of origin. Recently, the results from the LIBRETTO-001 and ARROW clinical trials demonstrated significant clinical benefits with selpercatinib and pralsetinib respectively, in NSCLC patients with RET gene fusions, with tolerable toxicity profiles. These studies also demonstrated that these RET-TKIs crossed the blood brain barrier with significant activity. As has been observed with other TKIs, the emergence of acquired resistance may limit long-term efficacy of these agents. Therefore, understanding the mechanisms of resistance is necessary for the development of strategies to overcome them.

## Introduction

Comprehensive genomic testing is now the standard of care in the management of metastatic non-small cell lung cancer (NSCLC). The goal of genomic testing is to identify common or uncommon actionable genomic alterations that impact therapeutic decision making. The NCCN guidelines recommends testing for the certain molecular and immune biomarkers in patients with metastatic NSCLC to assess eligibility for targeted therapy or immunotherapy.

Predictive biomarkers include gene fusions in *ALK, ROS1, NTRK*, and *RET*, sensitizing *EGFR* gene mutations, *BRAF* V600E point mutations, *MET* exon 14 skipping mutations and amplifications, PD-L1 expression, *ERBB2* mutations, and tumor mutational burden. Targeted therapies to these biomarkers have demonstrated greater clinical efficacy when compared to chemotherapy [1–3].

In NSCLC, chromosomal rearrangements (fusion) between the Rearranged during transfection (RET) gene and another domain, most commonly kinesin family 5B (KIF5B) and coiled coil domain containing-6 (CCDC6), lead to overexpression of the RET protein [4]. The RET fusion occurs in 1–2% of NSCLC, particularly in younger, non‐smoking patients with adenocarcinoma histology [5], and they appear to be associated with a high risk of metastasis to the brain [6]. In contrast, *KIF5B-RET* and *CCDC6-RET* fusion genes have been identified in 70 to 90% and 10 to 25% of tumors, respectively [7]. RET fusion are thought to be exclusive of *EGFR, ALK, KRAS* and *BRAF* mutations, suggesting that it has its own oncogenic driver potential [5]. A number of RET fusion inhibitors have recently been approved, while others are in clinical trials. Patients with *RET* fusions have minimal response to immunotherapy [8].

## Molecular biology of RET gene fusions

**Fig. 1:**
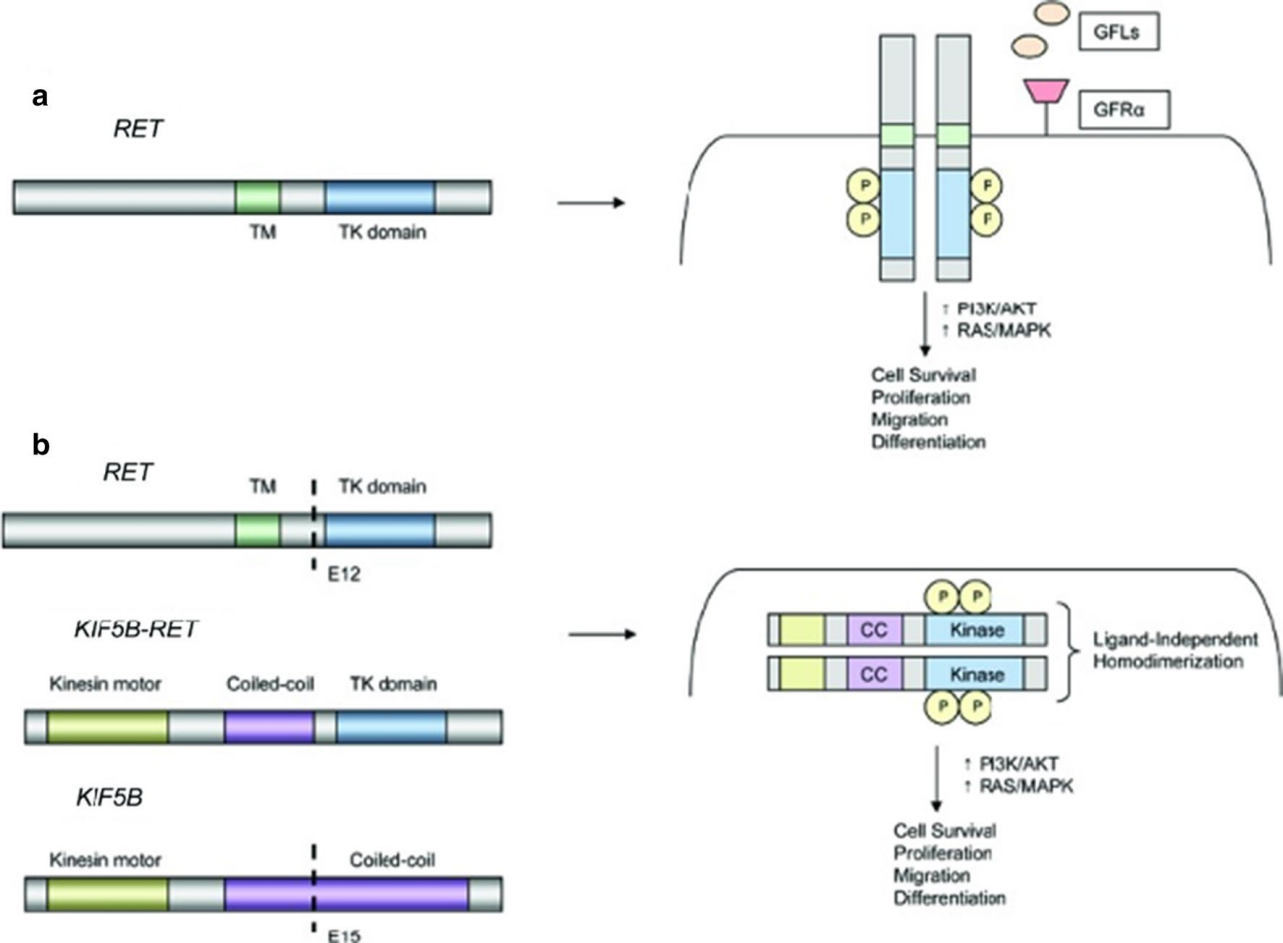
Mechanism of RET Gene rearrangements [12]. Models of RET rearrangements. **a** Schematic representation of the *RET* proto-oncogene (left). RET activation typically involves ligand binding, interactions with a coreceptor, and homodimerization leading to formation of a multiprotein complex (right). **b** Schematic representation of a *KIF5B-RET* fusion (left). The coiled-coil domain of KIF5B promotes ligand-independent homodimerization of RET, leading to constitutive activation of downstream growth signalling. License for reuse from John Wiley and Sons form Justin F. Gainor and Alice T. Shaw. Permission conveyed through Copyright Clearance Center, Inc. (License #: 4987820544353)

The *RET* gene is translated into a transmembrane receptor tyrosine kinase (RTK) with proto-oncogene properties. This RTK binds with various neurotrophic ligand-co-receptor complexes allowing adaptor and signaling protein to bind to RET intracellular tyrosine kinase residues that have undergone dimerization and autophosphorylation, leading to activation of downstream signaling pathways such as RAS/MAPK, PI3K/AKT, and JNK (Fig. 1a). *RET* fusions are caused by chromosomal rearrangements consisting of the juxtaposition of the C-terminal region of the RET protein with the N-terminal portion of another protein, leading to constitutive activation of the RET kinase [9]. The most common gene fusion partners are *KIF5B* and *CCDC6*, and less common fusion partners include *NCOA4, TRIM33, ZNF477P, ERCC1, HTR4*, and *CLIP1* [10]. *KIF5B* is the most common rearrangement observed in NSCLC, about 70% of RET-positive cases [11]. These rearrangements that produce chimeric fusion proteins can cause ligand-independent constitutive activation of RET, promoting cancer cell growth, proliferation, and survival [12] (Fig. 1b).

RET protein is comprised of three domains—an extracellular ligand-binding domain which includes four cadherin-like repeats and a cysteine-rich region, a hydrophobic transmembrane domain, and a cytoplasmic TK domain. Growth factors of the glial cell line-derived neurotrophic factor (GDNF) family comprise the multimolecular complex that binds to RET. These GDNF-family ligands (GFLs) bind to and activate RET when bound to GDNF-family receptor-a (GFRa) proteins. GFRas are ligand-binding co-receptors that lack intracellular or transmembrane domains and are anchored to the surface of the cell by glycosylphosphatidyl inositol (GPI)-linkage. Homodimeric GFLs activate the transmembrane RET TK by binding to different GPI-linked GFRa receptors with high affinity. When the ligand-GFRa complex binds to RET, homodimerization of RET and phosphorylation of tyrosine residues occur, resulting in subsequent intracellular signaling. RET activates various intracellular signaling cascades that regulate cell survival, proliferation, differentiation, migration, chemotaxis, and more, via several pathways including Ras/RAF and PI3K/AKT [13].

Mutations in *RET* have been implicated in the progression of several different disorders, including various solid tumors. For example, germline variants in *RET* result in decreased numbers of functional RET receptors on developing gut tissue, which leads to the failure of neuroblast migration and enteric nervous system development as observed in Hirschprung’s disease. Germline mutations that activate *RET* activity have been associated with multiple endocrine neoplasia 2 (MEN 2), which consists of three primary tumor types (medullary thyroid cancer, pheochromocytoma, and parathyroid hyperplasia or adenoma) [14].

## Diagnostic testing for RET fusions-tissue and liquid biopsies

*RET* fusions may be detected on tissue biopsies by various methods, including FISH, IHC, and reverse transcription PCR, but drawbacks to these approaches include the interrogation of limited numbers of gene partners and the inability to identify novel gene fusion partners, as well as weak staining patterns and reactivity for protein-dependent assays [10]. Next-generation sequencing (NGS) of DNA or RNA can interrogate multiple genes simultaneously, increasing the sensitivity of the assay to find these rare events. RNA sequencing can allow for a more comprehensive approach, as it not only identifies expressed fusion genes and discriminates splicing isoforms, but also provides quantification of fusion transcripts. RNAseq also allows for the detection of known and unknown expressed gene fusions as it does not rely on sequencing intronic regions that may harbor large repetitive sequences which are known to impair sequencing efficiency [15]. As tissue samples are limited, utilizing a comprehensive genomic analysis may be the most efficient method to detecting oncogenic driver mutations, including *RET* rearrangements. However, not all patients are able to receive comprehensive genomic profiling as up to 40% of tissue biopsies are not adequate for molecular testing [16, 17].

Liquid biopsies are a well-validated, FDA-approved molecular diagnostic tool that leverage circulating cell-free DNA (cfDNA) shed from advanced stage solid tumors, which can be interrogated for tumor-specific alterations utilizing hybrid-capture digital next-generation sequencing [18]. Numerous studies have demonstrated the utility of liquid biopsy to identify oncogenic driver mutations resulting in favorable clinical outcomes when patients are treated with targeted therapy [19–21]. An additional application of liquid biopsy is to detect acquired molecular mechanisms of resistance to targeted therapy, which can be missed if repeated tissue biopsies are not performed at disease progression [20, 22, 23]).

Several case reports have demonstrated the ability of liquid biopsy to detect RET rearrangements in NSCLC patients, who responded favorably to TKI. Perhaps even more impactful is the ability for liquid biopsy to detect acquired RET mutations that are acquired as resistance alterations to targeted therapy. These mutations include RET V804 gatekeeper mutation, solvent front mutations G810S/R/C, and acquired alterations in other genes such as EGFR, BRAF, PIK3CA, and others [21, 24, 25].

An analysis of over 32,000 plasma samples collected from advanced cancer patients was performed to elucidate the co-occurring *RET* alterations oncogenic signaling pathways identified in liquid biopsy. This study was the largest cancer cohort with somatic activating RET alterations and found that non-*KIF5B-RET* fusions contributed to anti-EGFR therapy resistance [26]. Thus, liquid biopsies have shown clinical utility in identifying oncogenic driver mutations for advanced NSCLC, as well as acquired resistance alterations.

## First-line treatment in patients with advanced disease

Earlier RET targeted agents were multi-targeted TKIs with indications in others solid tumors such as renal cell carcinoma, hepatocellular carcinoma, or thyroid cancer. Though these drugs inhibited the RET tyrosine kinase, they had limited potency for RET as they were not RET-specific inhibitors [27]. Selpercatinib (LOXO-292) is a novel, ATP-competitive, highly selective small-molecule inhibitor of RET kinase. Selpercatinib was also designed to penetrate the central nervous system (CNS) and has been shown in preclinical models to have antitumor activity in the brain [28]. LIBRETTO-001 is a study that enrolled 105 patients with advanced *RET* fusion–positive NSCLC who had previously received platinum-based chemotherapy and 39 previously untreated patients separately in a phase 1/2 trial of selpercatinib [29]. Of the 105 previously treated patients, 38 patients had CNS metastases at baseline, 11 of whom were deemed to have measurable lesions by RECIST version 1.1 (Table 1).

**Table 1:** Efficacy of Selpercatinib in LIBRETTO-001

*Prior platinum doublet (n = 105)*	
ORR (%)	68 (95% CI 58–76)
Medium DOR (months)	20.3 (95% CI 13.8–24.0)
Medium PFS (months)	18.4 (95% CI 12.9–24.9)
*Treatment naïve (n = 34)*	
ORR (%)	85 (95% CI 69–95)
Medium DOR (months)	NR
PFS (months)	NR
*Patients with measurable CNS metastasis (n = 11)*	
ORR (%)	91% (95% CI 59–100)

Most AEs were low grade. The most common adverse events of grade 3 or higher were hypertension (in 14% of the patients), an increased alanine aminotransferase level (in 12%), an increased aspartate aminotransferase level (in 10%), hyponatremia (in 6%), and lymphopenia (in 6%). Only 2% of patients discontinued selpercatinib due to AEs

NR, Not Reached; ORR, Objective Response Rates; DOR, Duration of Response; PFS, Progression-Free Survival

For the 39 patients who were previously untreated, neither the median duration of response nor the median progression-free survival had been reached at a median follow-up of 7.4 and 9.2 months, respectively. Selpercatinib had tolerable toxicity profile and most adverse events (AEs) were low grade. The most common AEs of grade 3 or higher were hypertension (in 14% of the patients), an increased alanine aminotransferase level (in 12%), an increased aspartate aminotransferase level (in 10%), hyponatremia (in 6%), and lymphopenia (in 6%). Only 2% of patients discontinued selpercatinib due to a drug-related adverse event (Table 2). On May 8, 2020 the Food and Drug Administration approved selpercatinib for NSCLC and Thyroid cancers with *RET* gene mutations or fusions. Also, in the NCCN guidelines (Version 2.2021), the NSCLC Panel recommends selpercatinib as a first-line or subsequent therapy option (category 2A; preferred) for patients with metastatic NSCLC who are positive for RET fusions [30].

**Table 2:** Selpercatinib safety overview

	TRAEs with selpercatinib (LOXO-292)	LIBRETTO-001 Safety Database* (N = 531)
	Any	Grade 3
Dry mouth	27	–
Diarrhea	16	1
Hypertension	18	8
AST increased	22	4
ALT increased	21	6
Fatigue	14	< 1
Constipation	11	< 1
Headache	7	< 1
Nausea	8	< 1
Peripheral edema	10	–
Creatinine increased	10	–

TRAEs, Treatment related adverse events

*Includes all tumor types (eg, NSCLC, MTC, Thyroid, etc.)

Pralsetinib (BLU-667) is also highly selective for the RET tyrosine kinase, have activity against multiple RET rearrangements, and have central nervous system (CNS) activity in mouse models [30–32]. Pralsetinib was investigated in a phase I/II ARROW trial, which enrolled patients with *RET* + NSCLC who were treated previously with platinum-based therapy and who were platinum naïve [33]. The recommended dose for phase II trials was 400 mg daily. At the time of the analysis 120 patients with RET + NSCLC were included, and 91 patients had received previous therapy with platinum-based therapy. The most common *RET* fusion partner was *KIF5B* in 79 patients (66%), followed by *CCDC6* in 16 patients (13%) [15, 34, 35] (Table 3).

**Table 3:** Efficacy of Pralsetinib in ARROW trial

	Overall (n = 116)	Prior platinum treatment (n = 80)	No prior systemic treatment (n = 26)	Measurable Brain Metastases (n = 9)
ORR, %	65 (5% CI 55–73)	61 (95% CI 50–72)	73 (95% CI 52–88)	55
DOR	NR	–	–	–
DCR, %	93 (87–97)	95 (95% CI 88–99)	88 (9% CI 70–98)	–

ORR, Objective Response Rates; DOR, Duration Of Response; DCR, Disease Control Rates. ORR was similar regardless of RET fusion partner, prior therapies, or central nervous system involvement. Overall, there were 7 (6%) completed responses, 4 (5%) in prior platinum patients and 3 (12%) in treatment naïve patients

Pralsetinib was well tolerated. Most adverse events (AEs) were low grades. The treatment-related grade ≥ 3 AEs observed in ≥ 5% of patients were neutropenia (*n* = 16, 13%), and hypertension (*n* = 12, 10%). Eight patients (7%) discontinued therapy due treatment-related AEs (Table 4). On September 4, 2020 the Food and Drug Administration approved pralsetinib for NSCLC with RET gene fusions. Also, in the NCCN guidelines (Version 2.2021), the NSCLC Panel recommends pralsetinib as a first-line or subsequent therapy option (category 2A; preferred) for patients with metastatic NSCLC who are positive for RET rearrangements [30].

**Table 4:** Pralsetinib safety overview

	TRAEs with Pralsetinib (BLU-667), %	ARROW safety population* (N = 354)
	Any	Grade ≥ 3
AST	31	2
Anemia	22	8
ALT increased	21	1
Constipation	21	1
Hypertension	20	10
Neutropenia	19	10
Diarrhea	14	1
WBC decreased	14	3
Dysgeusia	13	0
Creatinine increased	13	0
Neutrophil Count Decreased	13	4

^*^Includes all tumor types (eg, NSCLC, Thyroid, etc.)

## Mechanisms of resistance to RET fusion inhibitors

RET mutation-mediated resistance to multi-kinase inhibitors (MKIs) has been previously reported in single patients (e.g., *RET* V804M gatekeeper mutations and *RET* S904F). However, mechanisms underlying resistance to selective RET TKIs remain unknown [24, 25, 31]. Selective for RET TKIs show similar potency against wild-type RET and *RET* V804M/L in cellular assays. Furthermore, clinical activity has already been observed with selpercatinib in patients with medullary thyroid cancers harboring the *RET* V804M gatekeeper mutation [28].

Solomon et al. [24] noted that after a dramatic initial response to selpercatinib in a patient with *KIF5B-RET* NSCLC, analysis of circulating tumor DNA revealed emergence of *RET* G810R, G810S, and G810C mutations in the RET solvent front before the emergence of clinical resistance. Postmortem biopsy studies confirmed the presence of these mutations in multiple disease sites indicative of a common mechanism of resistance. They also described a second case of a heavily pretreated patient with *CCDC6-RET* fusion-positive NSCLC. He subsequently received a selective RET TKI with disease progression after an initial systemic and intracranial tumor response to selpercatinib. Sanger and next-generation sequencing analysis identified an acquired RET G810S mutation (and no other RET mutations) in malignant pleural cells, which was absent from pleural fluid collected immediately before selpercatinib treatment.

Although selective RET inhibitors are well tolerated and induce significant and durable tumor responses in heavily pretreated patients with RET-rearranged NSCLC, however, as has been seen with other selective TKIs, the emergence of acquired resistance may limit long-term efficacy.

## Discussion and conclusion

Comprehensive genomic testing is now the standard of care in the management of metastatic NSCLC. The goal of genomic testing is to identify actionable genomic alterations that inform therapeutic decision making. RET rearrangements were identified as oncogenic drivers in NSCLC, and are more common among younger patients, adenocarcinoma histology, and patients with a history of never smoking. The prevalence is estimated to be 1–2% among patients with adenocarcinoma histology. The most common rearrangement is between intron 11 of the *RET* gene and intron 15 of the *KIF5B* gene, and the next most frequent rearrangement is with the CCDC6 gene. *RET* fusions lead to constitutive activation of the RET tyrosine kinase and increased cell proliferation, migration, and survival [34].

Initial RET gene targeted agents were multi-kinase inhibitors (MKIs) such as vandetanib and cabozantinib that were indicated for other solid tumors such as medullary thyroid carcinoma, renal cell carcinoma, and hepatocellular carcinoma. Though these agents inhibited RET tyrosine kinase activity, their potency was limited because they were not RET-specific [34]. Data from studies of these agents in the NSCLC space were not encouraging. This gave impetus to the development of more specific and more potent RET TKIs. Selpercatinib (LOXO-292) and pralsetinib (BLU-667) are both second generation RET TKIs. Selpercatinib and pralsetinib have been shown to be efficacious and well tolerated due to their selectivity compared to MKIs in phases I/II clinical trials [15, 28–30, 33, 34]. Moreover, the excellent intracranial activity of selpercatinib and pralsetinib seen in these trials further provides a another advantage of these agents compared with vandetanib and cabozantinb that were associated with low CNS activity in RET fusion positive NSCLCs. As the use of selective RET TKIs becomes more widespread, it is inevitable for resistance to develop. Most acquired resistance mechanisms have been due to G810 solvent front mutations of the RET gene [36]. Alternatively, disease progression could develop due to upregulation of bypass tracks resulting in RET independent mechanisms of resistance. Therefore, it is imperative to obtain tissue or liquid biopsies for NGS when patients progress to determine the mechanisms of resistance.

Immune checkpoint inhibitors (ICIs) are now part of the standard of care for the treatment of metastatic NSCLC. However, studies suggest that most RET gene rearranged NSCLC have low PD-L1 expression and low TMB, and have inferior activity to ICIs [8, 37, 38]. In a retrospective study that included 551 patients with NSCLC, 16 patients had RET gene rearrangement [8]. Most of the patients had adenocarcinoma and were treated with the PD-1 inhibitors nivolumab or pembrolizumab. Patients were followed for a median of 16.1 months, and the ORR among patients with RET gene rearrangements was 12.7%, and progressive disease was observed in 75% of patients. The median OS was 18.4 months (95% CI, 7.0-NR), and median PFS was 3.4 months (95% CI, 1.7–6.2). These results suggest that immune checkpoint inhibitors (ICIs) should not be used as single agents in patients with RET gene rearranged NSCLC.

It has been demonstrated that pemetrexed-based chemotherapy regimen have modest activity in patients with *RET* rearranged NSCLC. Shen et al. [39] retrospectively evaluated 62 patients with stage IIIB/IV NSCLC and RET rearrangements, including 41 with *KIF5B-RET*, 15 with *CCDC6-RET*, and 6 with other rare fusion subtypes. Of the 40 patients who received first-line chemotherapy, the median PFS was significantly different between those receiving pemetrexed-based chemotherapy and those receiving other chemotherapy regimens (9.2 vs. 5.2 months; *P* = 0.007). The median PFS for patients with *KIF5B-RET* fusion and noneKIF5B-RET fusion was not significantly different statistically (7.8 vs. 11.2 months; *P* = 0.847). For second-line chemotherapy, a statistically significant difference was found between the chemotherapy regimens (4.9 vs. 2.8 months; *P* = 0.049). Survival follow-up data were available for 38 patients with advanced NSCLC. The median overall survival was 26.4 months. The overall survival of the patients with RET-rearranged NSCLC who had received pemetrexed-based chemotherapy versus no pemetrexed-based chemotherapy was 35.2 versus 22.6 months (*P* = 0.052). No difference in survival was observed between the patients with *KIF5B-RET* and none *KIF5B-RET* rearrangements. The efficacies and safeties among different strategies in the treatment of *RET* rearranged NSCLC are summarized (Table 5).

**Table 5:** Association of efficacy with treatment strategies in RET fusion positive NSCLC

Agent	ORR (%)	mPFS (mo)	mOS (mo)	Common Gr 3 or 4 AEs	Refs
Selpercatinib (first line)	85	NR		Hypertension, elevated AST, ALT,	[27]
Pralsetinib (first line)	73	NR		Anemia, hypertension, Neutropenia,	[32]
Vandetanib	18	4.5	11.6	Hypertension, diarrhea, rash, dry skin, QT prolongation	[41]
Cabozantinib	28	5.5	9.9	Elevated lipase, ALT, AST, thrombocytopenia, hypophosphatemia	[42]
CPI (Nivolumab or Pembrolizumab)	12.7	3.4 (95% CI 1.7–6.2)	18.4 (95% CI 7.0–NR)	NA	[8]
Platinum-Pemetrexed (first line)	50	9.2	26.4	NA	[37]

CPI, Checkpoint inhibitor; AST, Aspartate aminotransferase; ALT, Alanine aminotransferase; NA, Not available (these were retrospective studies focused on efficacy)

The emergence of non-*KIF5B-RET* fusion is a rare mechanism of EGFR TKI resistance, and appear to be more common with Osimertinib exposure than with first and second generation EGFR TKIs and may have an incidence of 4.9%. Rich et al. [26] reported six patients who acquired primarily, *CCDC6-RET* and *NCOA4*-*RET* fusions as mechanism of resistance to Osimertinib. Piotrowska et al. [36] reported two patients with similar findings where the T790M resistance clone was successfully suppressed with osimertinib while the *CCDC6-RET* fusion was detected at progression. In these two cases, combination therapy with osimertinib and selpercatinib was well-tolerated and led to rapid radiographic responses. Other ongoing clinical trials on the administration RET fusions inhibitors for NSCLC patients have been summarized (Table 6).

**Table 6:** *RET* gene fusions inhibitors in clinical trials [40]

Clinicaltrials.gov identifier	Study agent	Trial Phase	Trial description
NCT01639508	Cabozantinib	Phase II	Cabozantinib in Patients With RET Fusion-Positive Advanced Non-Small Cell Lung Cancer and Those With Other Genotypes: ROS1 or NTRK Fusions or Increased MET or AXL Activity
NCT04194944	Selpercatinib vs Carboplatin/Cisplatin + Pemetrexed ± Pembrolizumab	Phase III	A Study of Selpercatinib (LY3527723) in Participants With Advanced or Metastatic RET Fusion-Positive Non-Small Cell Lung Cancer (LIBRETTO-431)
NCT04268550	Selpercatinib	Phase II	Targeted Treatment for RET Fusion-Positive Advanced Non-Small Cell Lung Cancer (A LUNG-MAP Treatment Trial)
NCT04131543	Cabozantinib	Phase II	Phase II Study With Cabozantinib in Patients With RET Positive NSCLC (CRETA)
NCT03037385	Pralsetinib (BLU-667)	Phase I/II	Phase I/II Study of the Highly-selective RET Inhibitor, Pralsetinib (BLU-667), in Patients With Thyroid Cancer, Non-Small Cell Lung Cancer, and Other Advanced Solid Tumors (ARROW)
NCT04222972	Pralsetinib vs Carboplatin/Cisplatin + Pemetrexed ± Pembrolizumab or Carboplatin/Cisplatin Gemcitabine (Squamous histology)	Phase III	AcceleRET Lung Study of Pralsetinib for 1L RET Fusion-positive, Metastatic NSCLC
NCT04683250	TAS0953/HM06	Phase I/II	Study of RET Inhibitor TAS0953/HM06 in Patients With Advanced Solid Tumors With RET Gene Abnormalities ((MARGARET))
NCT03780517	BOS172738	Phase I	Safety, Efficacy, and Tolerability of BOS172738 in Patients With Advanced Rearranged During Transfection (RET) Gene-Altered Tumors
NCT03157128	LOXO-292 (Selpercatinib)	Phase I/II	Phase 1/2 Study of LOXO-292 in Patients With Advanced Solid Tumors, RET Fusion-Positive Solid Tumors, and Medullary Thyroid Cancer (LIBRETTO-001)
NCT04161391	TPX-0046	Phase I/II	Study of TPX-0046, A RET/SRC Inhibitor in Adult Subjects With Advanced Solid Tumors Harboring RET Fusions or Mutations

In summary, cfDNA NGS testing may be beneficial at identifying potentially actionable alterations in RET gene as well as resistance mechanisms that may be present following initial response of *RET* rearranged NSCLC to TKIs. cfDNA testing is noninvasive and simplifies studying the dynamics of response of *RET* rearranged NSCLC to TKIs and detects molecular disease progression which precedes radiographic disease progression.

## Data Availability

Not applicable as no datasets were generated or analyzed.

## References

[1] Mazieres J, Zalcman G, Crino L (2015). Crizotinib therapy for advanced lung adenocarcinoma and a ROS1 rearrangement: results from the EUROS1 cohort. J Clin Oncol.

[2] Sholl LM, Aisner DL, Varella-Garcia M (2015). Multi-institutional oncogenic driver mutation analysis in lung adenocarcinoma: the lung cancer mutation consortium experience. J Thorac Oncol.

[3] Planchard D, Besse B, Groen HJM (2016). Dabrafenib plus trametinib in patients with previously treated BRAF(V600E)-mutant metastatic non-small cell lung cancer: an open-label, multicentre phase 2 trial. Lancet Oncol.

[4] Santoro M, Melillo R, Carlomagno F, Fusco A, Vecchio G (2006). Molecular mechanisms of RET activation inhuman cancer. Ann N Y Acad Sci.

[5] Takeuchi K, Soda M, Togashi Y, Suzuki R, Sakata S, Hatano S (2012). RET, ROS1 and ALK fusions in lung cancer. Nat Med.

[6] Drilon A, Lin JJ, Filleron T (2018). Frequency of brain metastases and multikinase inhibitor outcomes in patients with RET-rearranged lung cancers. J Thorac Oncol.

[7] Sarfaty M, Moore A, Neiman V (2017). RET fusion lung carcinoma: response to therapy and clinical features in a case series of 14 patients. Clin Lung Cancer.

[8] Mazieres J, Drilon A, Lusque A (2019). Immune checkpoint inhibitors for patients with advanced lung cancer and oncogenic driver alterations: results from the IMMUNOTARGET registry. Ann Oncol.

[9] Tsuta K, Kohno T, Yoshida A (2014). *RET*-rearranged non-small-cell lung carcinoma: a clinicopathological and molecular analysis. Br. J Cancer.

[10] Go H, Kim YT (2013). Diagnostic method for the detection of KIF5B-RET transformation in lung adenocarcinoma. Lung Cancer.

[11] Benayed R, Offin M, Mullaney K (2019). High yield of RNA sequencing for targetable kinase fusions in lung adenocarcinomas with no mitogenic driver alteration detected by DNA sequencing and low tumor mutation burden. Clin Cancer Res.

[12] Gainor JF, Shaw AT (2013). Novel targets in non-small cell lung cancer: ROS1 and RET fusions. Oncologist..

[13] Arighi E, Borrello MG, Sariola H (2005). RET tyrosine kinase signaling in development and cancer. Cytokine Growth Fact. Rev..

[14] Li AY (2019). RET fusions in solid tumors. Cancer Treat. Rev..

[15] Bruno R, Fontanini G (2020). Next generation sequencing for gene fusion analysis in lung cancer: a literature review. Diagnostics.

[16] Meric-Bernstam F, Brusco L, Shaw K (2015). Feasibility of large-scale genomic testing to facilitate enrollment onto genomically matched clinical trials. J Clin Oncol.

[17] Aggarwal C, Thompson JC, Black TA (2019). Clinical implications of plasma-based genotyping with the delivery of personalized therapy in metastatic non-small cell lung cancer. JAMA Oncol.

[18] Odegaard JI, Vincent JJ, Mortimer S (2018). Validation of a plasma-based comprehensive cancer genotyping assay utilizing orthogonal tissue- and plasma-based methodologies. Clin. Cancer Res.

[19] Reckamp KL, Patil T, Kirtane K (2020). Duration of targeted therapy in patients with advanced none-small-cell lung cancer identified by circulating tumor DNA analysis. Clin Lung Cancer.

[20] Mack PC (2020). Spectrum of driver mutations and clinical impact of circulating tumor DNA analysis in non-small cell lung cancer: analysis of over 8000 cases. Cancer.

[21] Helman E, Nguyen M, Karlovich CA (2018). Cell-free DNA next-generation sequencing prediction of response and resistance to third-generation EGFR inhibitor. Clin Lung Cancer.

[22] McCoach CE, Blakely CM, Banks KC (2018). Clinical utility of cell-free DNA for the detection of ALK fusions and genomic mechanisms of ALK inhibitor resistance in non-small cell lung cancer. Clin Cancer Res.

[23] Thompson JC, Yee SS, Troxel AB (2016). Detection of therapeutically targetable driver and resistance mutations in lung cancer patients by next-generation sequencing of cell-free circulating tumor DNA. Clin Cancer Res.

[24] Solomon BJ, Tan L, Lin JJ (2020). RET solvent front mutations mediate acquired resistance to selective RET inhibition in RET-driven malignancies. J Thorac Oncol.

[25] Dagogo-Jack I, Stevens SE, Lin JJ (2018). Emergence of a *RET* V804M gatekeeper mutation during treatment with vandetanib in *RET*-rearranged NSCLC. J Thoracic Oncol.

[26] Rich TA, Reckamp KL, Chae YK (2019). Analysis of cell-free DNA from 32,989 advanced cancers reveals novel co-occurring activating RET alterations and oncogenic signaling pathway aberrations. Clin Cancer Res.

[27] Drilon A, Hu ZI, Lai GGY (2018). Targeting RET-driven cancers: lessons from evolving preclinical and clinical landscapes. Nat Rev Clin Oncol.

[28] Subbiah V, Velcheti V, Tuch BB (2018). Selective RET kinase inhibition for patients with RET-altered cancers. Ann Oncol.

[29] Drilon A, Oxnard GR, Tan DSW (2020). Efficacy of selpercatinib in *RET* fusion-positive non-small-cell lung cancer. N Engl J Med.

[30] NCCN Guidelines Version 2.2021. Non-small cell lung cancer. Accessed on December 29, 2020. http://www.nccn.org.

[31] Evans E, Hu W, Cao F, et al. BLU-667 demonstrates robust activity in RET fusion- driven intracranial tumor models. In: The 2019 World conference on lung cancer, Barcelona, Spain, 7–10 September 2019. Abstract P2.03–44.

[32] Gainor JF, Lee DH, Curigliano G (2019). Clinical activity and tolerability of BLU-667, a highly potent and selective RET inhibitor, in patients (pts) with advanced RET-fusion+ non-small cell lung cancer (NSCLC). J Clin Oncol.

[33] Gainor JF, Curigliano G, Kim D-W (2020). Registrational dataset from the phase I/II ARROW trial of pralsetinib (BLU-667) in patients (pts) with advanced RET fusion+ non-small cell lung cancer (NSCLC) [abstract]. J Clin Oncol.

[34] Stinchcombe TE (2020). Current management of *RET*rearranged non-small cell lung cancer. Ther Adv Med Oncol..

[35] Nakaoku T, Kohno T, Araki M (2018). A secondary RET mutation in the activation loop conferring resistance to vandetanib. Nat Commun.

[36] Piotrowska Z, Isozaki H, Lennerz JK (2018). Landscape of acquired resistance to osimertinib in EGFR-mutant NSCLC and clinical validation of combined EGFR and RET inhibition with osimertinib and BLU-667 for acquired RET fusion clinic. Cancer Discov.

[37] Offin M, Guo R, Wu S (2019). Immunophenotype and response to immunotherapy of *RET*-rearranged lung cancers. JCO Precis Oncol.

[38] Lee J, Ku BM, Shim JH (2020). Characteristics and outcomes of *RET*-rearranged Korean non-small-cell lung cancer patients in real-world practice. Jpn J Clin Oncol.

[39] Shen T (2020). Association between RET fusions and efficacy of pemetrexed-based chemotherapy for patients with advanced NSCLC in China: a multicenter retrospective study. Clin Lung Cancer.

[40] www.clinicaltrials.gov. Accessed on January 16, 2021.

[41] Lee SH, Lee JK, Ahn MJ (2017). Vandetanib in pretreated patients with advanced non-small cell lung cancer-harboring RET rearrangement: a phase II clinical trial. Ann Oncol.

[42] Drilon A, Rekhtman N, Arcila M (2016). Cabozantinib in patients with advanced RET- rearranged non-small-cell lung cancer: an open-label, single-centre, phase 2, single-arm trial. Lancet Oncol.

